# Circulating Th17 T cells at treatment onset predict autoimmune toxicity of PI3Kδ inhibitors

**DOI:** 10.1038/s41408-023-00788-9

**Published:** 2023-02-02

**Authors:** Deepti Gadi, Stephen P. Martindale, Pui Yan Chiu, Jasneet Khalsa, Pei-Hsuan Chen, Stacey M. Fernandes, Zixu Wang, Svitlana Tyekucheva, John-Hanson Machado, David C. Fisher, Philippe Armand, Matthew S. Davids, Scott Rodig, Barbara Sherry, Jennifer R. Brown

**Affiliations:** 1 Department of Medical Oncology, Dana-Farber Cancer Institute, Boston, MA USA; 2 Department of Medicine, Harvard Medical School, Boston, MA USA; 3 Center for Immunology & Inflammation, Institute of Molecular Medicine, The Feinstein Institutes for Medical Research, Northwell Health, Manhasset, NY USA; 4 Center for Immuno-Oncology, Dana-Farber Cancer Institute, Boston, MA USA; 5 Department of Data Sciences, Dana-Farber Cancer Institute, Boston, MA USA; 6 Department of Molecular Medicine, Donald and Barbara Zucker School of Medicine at Hofstra/Northwell, Hempstead, NY USA

**Keywords:** Translational research, Chronic lymphocytic leukaemia

## Abstract

PI3Kδ inhibitors are approved for the therapy of B cell malignancies, but their clinical use has been limited by unpredictable autoimmune toxicity, despite promising efficacy and evidence that toxicity is associated with improved clinical outcomes. Prior phenotypic evaluation by CyTOF has identified increases in activated CD8 T cells with activation of Th17 T cells, as well as decreases in Tregs, particularly in patients with toxicity. Here we sought to further understand the effects of idelalisib and duvelisib in vitro, and demonstrate that both idelalisib and duvelisib can inhibit T cell proliferation as well as Th1 and Treg differentiation in vitro, while promoting Th2 and Th17 differentiation. We further demonstrate directly using intracellular flow cytometry that autoimmune toxicity in patients is associated with higher absolute numbers of CD4 and CD8 T cells with Th17 differentiation in peripheral blood prior to therapy, and that gastrointestinal tissues from patients with active autoimmune complications of PI3Kδ inhibitors show infiltration with Th17^+^ T cells. These same tissues show depletion of Tregs as compared to CLL patients without toxicity, suggesting that loss of Tregs may be permissive for Th17 activation to lead to autoimmune toxicity. Clinical trials to restore this balance are warranted.

## Introduction

Despite recent advances, chronic lymphocytic leukemia (CLL) remains incurable. Although targeted inhibitors have transformed CLL therapy [1], patients are increasingly relapsing after both BTK inhibitors and the BCL-2 inhibitor venetoclax. The use of phosphatidylinositol 3 kinase (PI3K) inhibitors, a third class of highly active agent, has been limited by their autoimmune toxicities [2, 3], which are worse in a frontline setting, despite promising efficacy [2, 4]. Understanding and targeting the mechanism of PI3Kδ-associated toxicity could allow us to more fully incorporate these agents into the CLL treatment paradigm and extend the duration of benefit from currently available therapies.

Approved PI3K inhibitors in CLL target the PI3Kδ isoform, which is important for B cell development, activation and migration [5, 6]. More recent data demonstrate that PI3Kδ inhibition also impacts T cells, disrupting Treg stability more than T effector function and promoting anticancer immunity [7]. A mouse with a kinase dead PI3Kδ develops colitis, similar to patients treated with PI3Kδ inhibitors, but also resists the development of leukemia and can acquire protective anti-cancer immunity [8]. The PI3Kγ isoform is also expressed predominantly in leukocytes and modulates macrophage and T cell function in the CLL microenvironment [9]. Mice lacking PI3Kγ show increased CD8 T cell activation and cytotoxicity due to transcriptional activation of NFκB and inhibition of C/EBPβ in macrophages within the microenvironment [9].

The first in class PI3K inhibitor idelalisib is specific for PI3Kδ [6], while duvelisib inhibits both PI3Kδ and PI3Kγ [10–12]. Idelalisib was approved by the US Food and Drug Administration (FDA) in 2014 in combination with rituximab for relapsed/refractory CLL patients in whom rituximab would be appropriate therapy, but is not widely used due to toxicities that include autoimmune colitis, pneumonitis, and transaminitis [2, 3, 13]. Duvelisib was approved in 2018, also for CLL after at least two prior therapies [14], but has had similar toxicity [14]. The preclinical data suggest that these two drugs of different isoform specificities may differ in the mechanism of autoimmune toxicity, but data to date remain limited [15, 16].

Immune-related adverse events (irAEs) caused by PI3Kδ inhibitors can be severe [2]. These irAEs were particularly severe in an investigator-sponsored phase 2 trial that we conducted in untreated CLL patients, testing idelalisib with a 2-month lead-in prior to ofatumumab [2, 17]. The fulminant toxicity observed in this study was primarily transaminitis, which we have previously shown to be autoimmune [2]. Based on this study we showed that irAEs are more common in younger, less heavily pretreated patients, and in those with mutated immunoglobulin heavy chain variable regions (IGHV) [2]. A subsequent analysis of all fully enrolled Gilead-sponsored, phase 2 and 3 studies confirmed that risk was greater in younger, previously untreated patients [18]. More recently, we conducted a phase 1/1b clinical trial in young fit CLL patients in which we combined duvelisib with fludarabine cyclophosphamide rituximab (FCR) therapy, with the goal of achieving a high rate of undetectable MRD (uMRD). The regimen was highly effective, with an 88% overall response rate (ORR) and 66% uMRD rate in bone marrow [4]. While the therapy was generally well tolerated, irAEs again included transaminitis (28% grade 3–4), arthritis (9% grade 2), and colitis (6% grade 2–3).

Our initial results evaluating T cell alterations in patients treated with PI3Kδ inhibitors demonstrated that T regulatory cells (Tregs) decreased early after idelalisib initiation, particularly in patients with toxicity [2, 19]. Our initial mass cytometry by time-of-flight (CyTOF) investigations suggested that the Th17 pathway was activated in patients with toxicity [19]. We therefore undertook these studies to assess the impact of both idelalisib and duvelisib on T cells in vitro, and to directly evaluate the Th17 pathway in patient samples from these trials, both in peripheral blood and tissues. Direct production of IL-17A, IL-17F and IFNγ by T cells most closely reflects the immediate capacity to trigger inflammation, so we evaluated the presence of these cytokines intracellularly within T cells. Our results demonstrate that higher absolute numbers of circulating Th17s at treatment initiation are associated with development of toxicity, and that gastrointestinal tissues affected by PI3K autoimmune toxicity show increased infiltration by T cells with a Th17 phenotype, as well as a reduction in Tregs. These results implicate the Th17 pathway in the autoimmune toxicity of PI3Kδ inhibitors and suggest a target for treatment.

## Patients and methods

### CLL sampling

CLL patient samples were obtained during two clinical trials or from an associated CLL tissue bank. All patients provided written informed consent to the local IRB approved protocol. The first trial was an investigator-sponsored phase 2 trial of idelalisib with ofatumumab, in which patients received two months of idelalisib prior to initiation of ofatumumab [17]. Both the autoimmune toxicity [2] and the clinical outcomes of this trial [17] have been reported. The second trial was an investigator-sponsored phase 1/1b trial combining duvelisib with FCR, for which the clinical outcomes [4] and correlative immune studies [15] have been reported. Peripheral blood mononuclear cells (PBMCs) were banked serially and at the time of emergent autoimmune toxicity. Patients were selected for study based on sample availability in relation to timing of toxicity and prior to initiation of steroids. Fewer patients on the idelalisib study were available for study at cycle 5 due to the high rate of steroid treatment and/or drug discontinuation. Patient characteristics are detailed in Supplementary Table 1. Pathology specimens were obtained in the course of routine clinical care for both patients with toxicity and controls, and were obtained for this evaluation retrospectively (Supplementary Table 2). Patients on both studies were characterized by grade of toxicity, with a particular focus on grade 3 or higher irAEs occurring in the first 3 months of therapy (transaminitis and colitis), as compared to later or no toxicity. This choice allowed us to focus on the most severe toxicities, which were early, and to keep the patient population as uniform as possible, i.e., maintained continuously on drug. Toxicities were graded according to the Common Terminology Criteria for Adverse Events version 4.03 [4].

### Flow cytometric analysis of IL-17A, IL-17F, and IFNγ in PBMCs ex vivo

For detection of intracellular IL-17A, IL-17F, and IFNγ, freshly thawed PBMC suspensions from CLL patients in the studies described above were thawed in 1× PBS containing 100 µg/mL DNase and 5 µM MgCL_2_, washed, counted, resuspended at 3 × 10^6^ cells per mL and stimulated for 3 h with 10 ng/mL phorbol-12-myristate-13-acetate (PMA) and 250 ng/mL ionomycin in the presence of monensin (BD Biosciences, San Jose, CA). Stimulated cells were centrifuged at 1200 × *g* for 7 min at 10 °C, washed, and then surface stained by incubating cells for 30 min at room temperature in the dark with anti-human mAbs: anti-CD3-APC-H7, -CD4-V450, -CD8-V500, and -CD19-APC (all BD Biosciences). Cells were subsequently fixed and permeabilized using Cell Fixation/Permeabilization Kit (BD Biosciences) and stained intracellularly with IL-17A PerCP Cy5.5 (BD Biosciences), IL-17F PE (eBioscience Inc.) and IFNγ AlexaFluor 488 (eBioscience Inc.) for 30 min according to the manufacturer’s instructions. Samples were acquired on Fortessa (BD Biosciences) and analyzed using the FlowJo software (version 8.8.6). Intracellular flow cytometry gating strategy used to determine the percentages of CD4 and CD8 cells positive for IL-17A and IL-17F is illustrated in Supplementary Fig. 1. The percentages of CD4 and CD8 cells positive for IFNγ were determined in a similar manner, as illustrated in Supplementary Fig. 2. The absolute number of CD4 and CD8 cells expressing IL-17A, IL-17F and IFNγ in blood (per mm^3^) for each patient was determined according to the formula: [ALC (per mm^3^)] × [% CD3-positive MNC] × [% CD4-positive CD3 cells] × [%IL-17-positive CD4 cells]. ALC means absolute lymphocyte count and MNC means mononuclear cells.

### Multiplex immunofluorescence

Multiplex immunofluorescent staining was performed on BOND RX fully automated stainers (Leica Biosystems), as previously described [20, 21]. Tissue sections of 5-μm thick FFPE were baked for 3 h at 60 °C before loading into the BOND RX. Slides were deparaffinized (BOND DeWax Solution, Leica Biosystems) and rehydrated with a series of graded ethanol to deionized water. Antigen retrieval was performed in BOND Epitope Retrieval Solution 1 or 2 (ER1 for all antigens except ER2 for RORγT, Leica Biosystems) at pH 6 for 10 min at 98 °C. Deparaffinization, rehydration, and antigen retrieval were all preprogrammed and executed by the BOND RX. Next, slides were serially stained with antibodies, incubation time per antibody was 40 min. Subsequently, anti-rabbit Polymeric Horseradish Peroxidase (Poly-HRP, BOND Polymer Refine Detection Kit, Leica Biosystems) was applied as a secondary label with an incubation time of 10 min. Signal for antibody complexes was labeled and visualized by their corresponding Opal Fluorophore Reagents by incubating the slides for 5 min. The same process was repeated for the following antibodies/fluorescent dyes. Slides were mounted with Prolong Diamond Anti-fade mounting medium (#P36965, Life Technologies) and stored in a light-proof box at 4 °C prior to imaging. The target antigens, antibody clones, and dilutions for markers included in this report are listed in Table 1.

**Table 1:** Target antigens, antibody clones, and dilutions for markers used.

Antigen	Antibody dilution	Clone	Manufacturer	Opal Fluorochrome
CD3	1:1000	poly	Dako	620
CD8	1:7000	C8/144B	Dako	540
CD4	1:250	4B12	Dako	520
FOXP3	1:2000	206D	Biolegend	570
Granzyme B	1:100	Grb-7	Dako	690
RORγT	1:150	6F3.1	BioCare Medical	650

### Statistics

Statistics were performed using GraphPad Prism software version 9.3.1. Data are summarized as median with interquartile range and statistical significance was determined using the Mann–Whitney *U*-Test. *p* values <0.05 were considered statistically significant.

Please see Supplementary Methods for additional methods.

## Results

### Impact of idelalisib and duvelisib on T cells in vitro

**Fig. 1:**
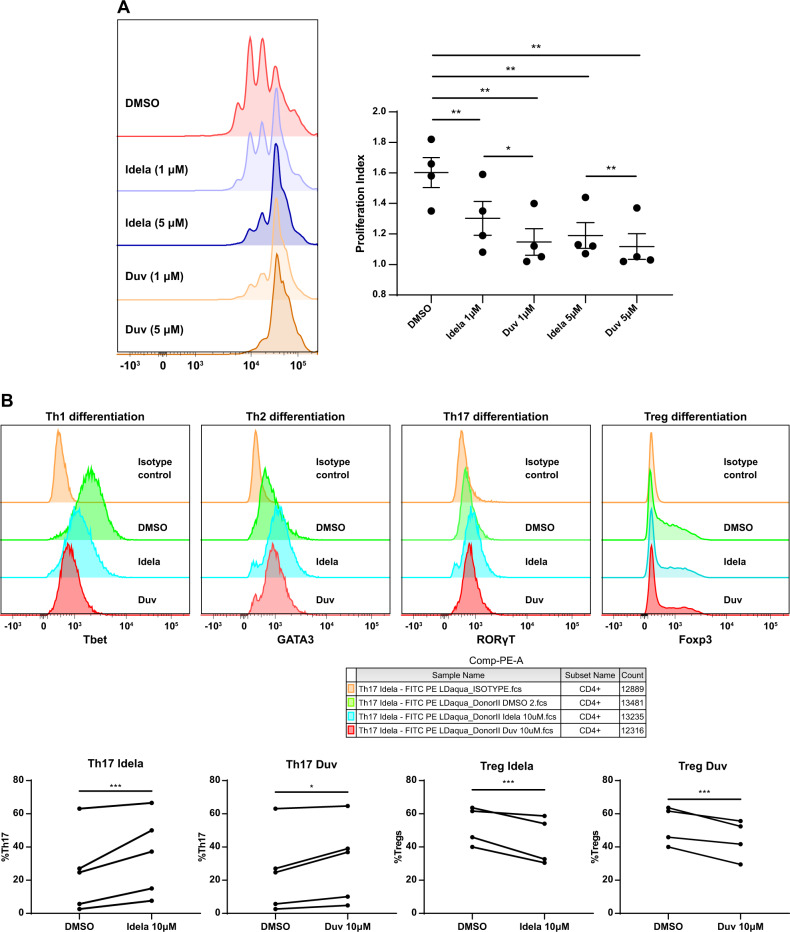
In vitro effects of idelalisib and duvelisib on T cell proliferation and differentiation. **A** Impact of the indicated concentrations of idelalisib (Idela) and duvelisib (Duv) on CD3 T cell proliferation as measured by Cell Trace Violet dye. Proliferation index was quantified using Modfit. **B** In vitro effects of idelalisib and duvelisib at 10 μM on differentiation of naive CD4^+^ T cells to Th1 (T-bet), Th2 (GATA-3), Th17 (RORγT), and Treg (FOXP3) phenotypes. *p ≤ 0.05; **p ≤ 0.01; ***p ≤ 0.001.

Given our prior work demonstrating the impact of idelalisib and duvelisib on Tregs in vivo [2, 15], as well as data pointing toward enhanced Th17 differentiation [16, 22], we evaluated the effect of each drug on T cell proliferation and differentiation in vitro. First, we assessed the effect of each drug on CD3 T cell proliferation in vitro by flow cytometry using Cell Trace Violet dye dilution assays. We found that 1 µM of either idelalisib or duvelisib significantly reduced the proliferation index of CD3 T cells in response to CD3/CD28 bead stimulation (Fig. 1A), demonstrating effects on proliferation of both CD4 and CD8 T cell subsets. Interestingly duvelisib led to a greater reduction in proliferation index compared to idelalisib at both 1 and 5 μM (Fig. 1A), likely related either to its greater potency against the delta isoform [23] or to its additional activity against the gamma isoform.

We also assessed the impact of idelalisib and duvelisib on in vitro CD4 T cell differentiation into T helper subsets by measuring the effects of each drug on CD3/CD28 induced differentiation of naive CD4^+^ T cells into Th1, Th2, Treg and Th17 cells. With either duvelisib or idelalisib treatment in vitro, we observed a decrease in Th1 differentiation as measured by T-bet expression, a decrease in Treg differentiation as measured by Foxp3 expression, and an increase in Th17 differentiation as measured by RORγT expression in T cells (Fig. 1B and Supplementary Fig. 3). Th2 differentiation was increased by idelalisib without a significant effect of duvelisib. These data are consistent with the changes we have previously seen in vivo with CyTOF analysis of CLL patient PBMCs [15, 16, 22].

### Direct assessment of Th17 pathway with idelalisib

**Fig. 2:**
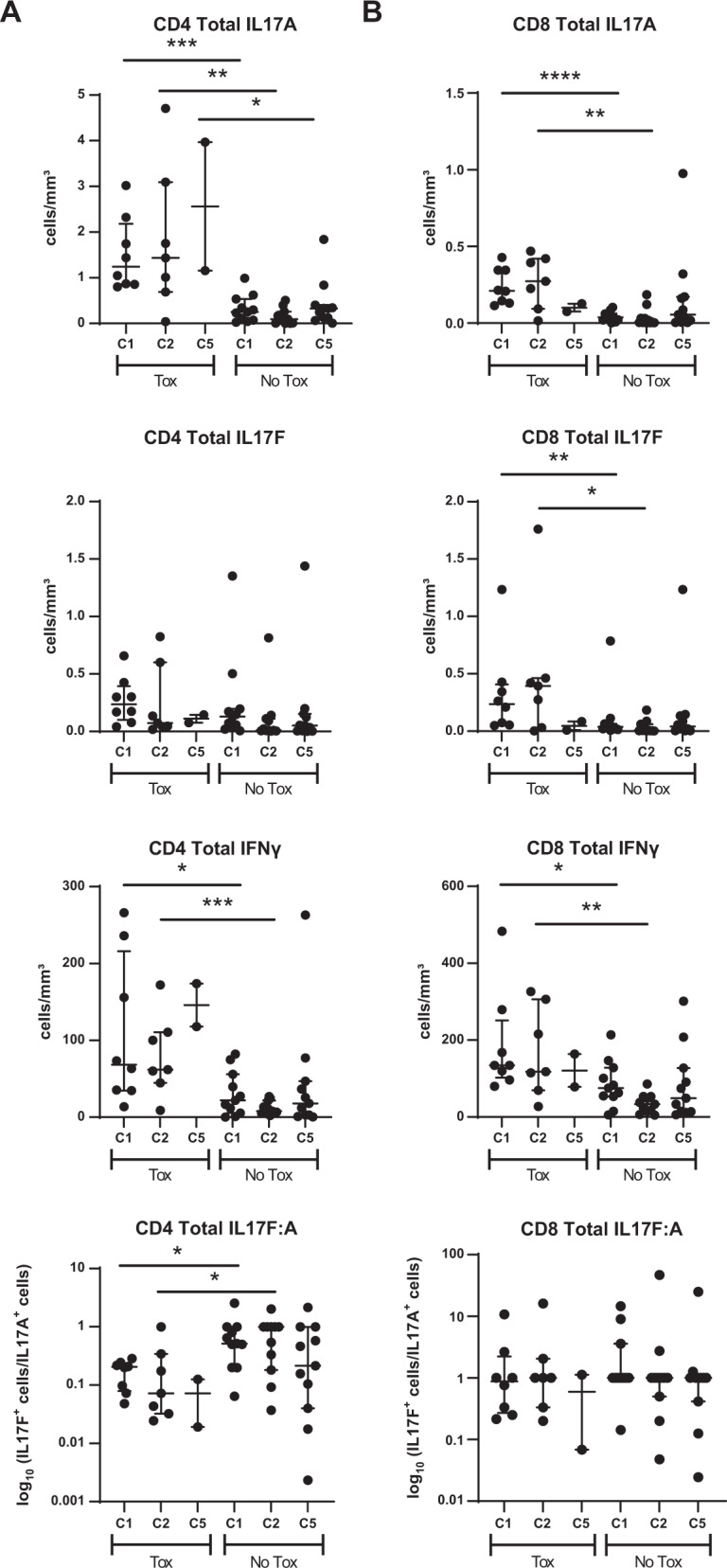
Intracellular flow cytometry measurements of IL-17A, IL-17F and IFNγ in CD4 and CD8 T cells treated with idelalisib. **A** Absolute number of CD4 T cells positive for the indicated cytokine at C1 (prior to treatment), C2 and C5, comparing patients with early toxicity (Tox) to either delayed or no toxicity (No Tox). The bottom panel is the ratio of IL17F:A. **B** Absolute number of CD8 T cells positive for the indicated cytokine at C1 (prior to treatment), C2 and C5, comparing patients with early toxicity (Tox) to either delayed or no toxicity (No Tox). The bottom panel is the ratio of IL17F:A. *p ≤ 0.05; **p ≤ 0.01; ***p ≤ 0.001; ****p ≤ 0.0001.

**Fig. 3:**
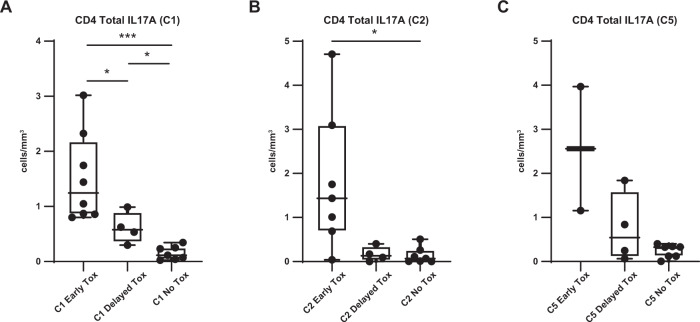
Absolute number of CD4^+^ Total IL-17A^+^ cells treated with idelalisib is increased in patients with early Tox compared to Delayed or No Tox. **A**–**C** Absolute number of CD4^+^ Total IL17A^+^ cells, comparing patients with early toxicity to delayed toxicity to no toxicity. **A** At C1 prior to therapy. **B** At C2. **C** At C5. *p ≤ 0.05; ***p ≤ 0.001.

To directly confirm the in vitro differentiation effects of idelalisib and duvelisib, we assessed the intracellular expression of IL-17A, IL-17F and IFNγ in CD4 and CD8 T cells isolated from peripheral blood of patients treated with each of these drugs, in relation to whether or not those patients developed severe autoimmune toxicity. For idelalisib, we evaluated patients treated on our frontline study of idelalisib with ofatumumab, in which 54% of patients developed grade 3–4 autoimmune hepatotoxicity at a median of 4 weeks on idelalisib [2]. Patients who developed this early severe hepatotoxicity were considered to have early toxicity in this study, which was definitely autoimmune [2] (*N* = 8 studied). An additional 25% patients developed only later toxicity, approximately cycle 3 or after, and the toxicity in these patients was less fulminant, behaving more similarly to what has been described in the relapsed/refractory setting [2, 3, 24]; this group was considered to have delayed toxicity (*N* = 4). Seven patients with no toxicity were studied for comparison (*N* = 7). We assessed PBMCs serially drawn at baseline (C1), after one month on therapy (C2; time of severe early toxicity when it occurred), and after four months on therapy (C5; time of delayed toxicity when it occurred). Given limited numbers of evaluable patients, for the primary statistical analysis, we combined delayed with no toxicity, to compare to early toxicity. As shown in Fig. 2, the most dramatic increase in toxicity was seen in patients in whom the absolute numbers of circulating CD4 cells positive for IL17A and IFNγ were high relative to patients with delayed or no toxicity (Fig. 2A). Furthermore, patients with toxicity showed significantly higher IL-17A- and IFNγ-positive CD4 cells at baseline as well as at C2 and C5. This resulted in a significant shift in the IL17F:IL17A ratio in CD4 T cells (Fig. 2A, bottom panel). Similar results were also seen with intracellular IL17A and IFNγ in CD8 T cells, but in the CD8 cells, greater differences were also seen in IL17F (Fig. 2B). Similar results were seen when evaluating the percentage of Th17-positive cells rather than the absolute number (Supplementary Fig. 4A). We further evaluated intracellular IL-17A in CD4 T cells separately in all three toxicity groups (Fig. 3), and show a graded effect, with the highest numbers in patients with early severe toxicity, followed by those with delayed toxicity, and the lowest numbers in those without toxicity. Interestingly, patients with delayed toxicity appear to show an increase in IL-17A in CD4 T cells at cycle 5, closer to the time of delayed toxicity, albeit with few evaluable patients.

In our prior work, we demonstrated that CLL patients with a mutated IGHV had a higher risk for early severe toxicity with idelalisib [2]. Prior work has also shown that CLL patients with a mutated IGHV have higher absolute numbers of circulating Th17s [25]. We therefore investigated whether higher Th17 activation was seen in patients with mutated IGHV and found that total IL-17A was elevated in CD4s and CD8s at all timepoints in patients with mutated IGHV, consistent with prior work (Supplementary Fig. 5A). Total IL17F and IFNγ in CD4s and CD8s trended higher at all timepoints, with significance at one (Supplementary Fig. 5A). These findings suggest that the increased risk of irAEs associated with mutated IGHV may be related to the association of mutated IGHV and increased circulating Th17s at baseline.

### Direct assessment of Th17 pathway with duvelisib

**Fig. 4:**
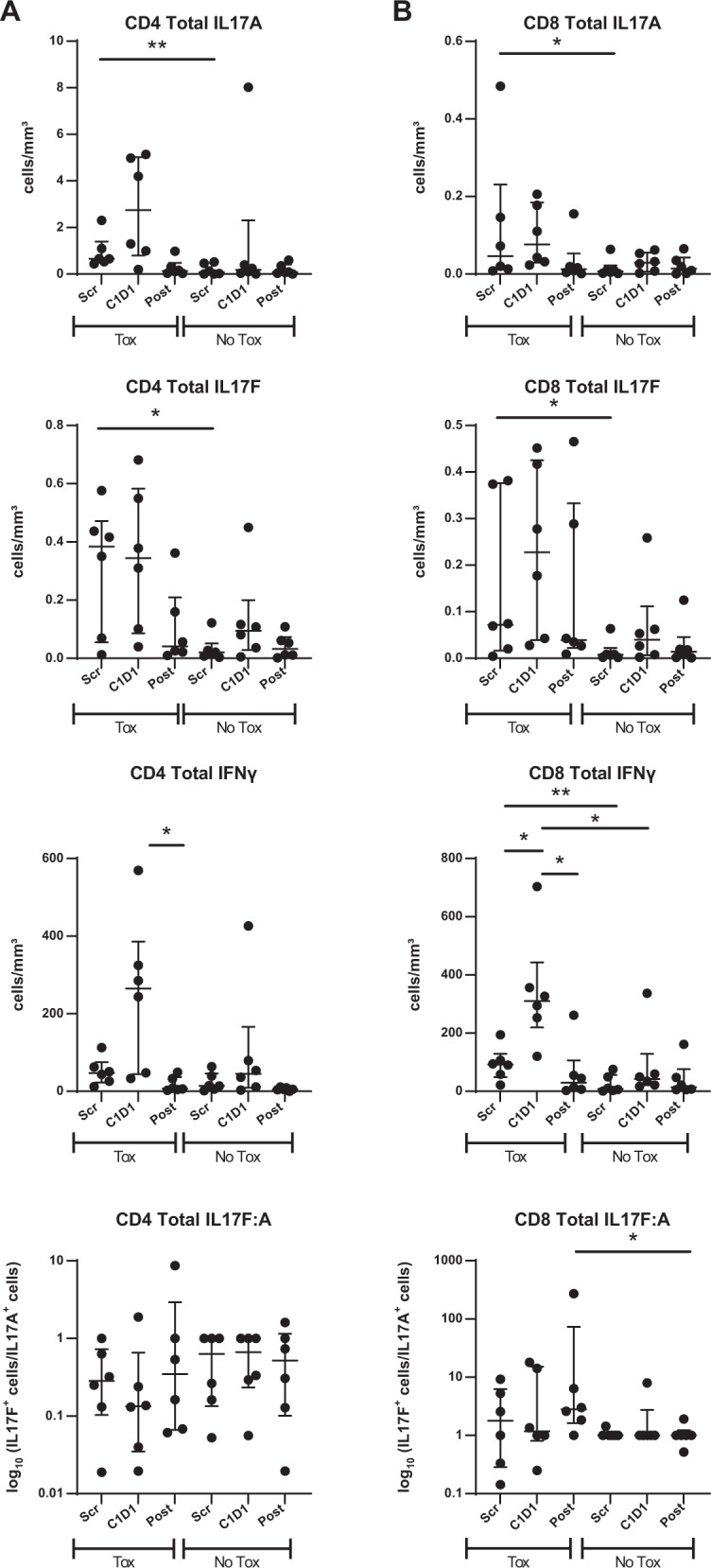
Intracellular flow cytometry measurements of IL-17A, IL-17F and IFNγ in CD4 and CD8 T cells treated with duvelisib. **A** Absolute number of CD4 T cells positive for the indicated cytokine at Scr (screen timepoint), C1D1 (7 days on duvelisib prior to FCR) and either 2 or 6 months post FCR, comparing patients with early toxicity (Tox) to either delayed or no toxicity (No Tox). The bottom panel is the ratio of IL17F:A. **B** Absolute number of CD8 T cells positive for the indicated cytokine at Scr (screen timepoint), C1 (7 days on duvelisib prior to FCR) and either 2 or 6 months post FCR, comparing patients with early toxicity (Tox) to either delayed or no toxicity (No Tox). The bottom panel is the ratio of IL17F:A. *p ≤ 0.05; **p ≤ 0.01.

We were then interested to determine whether similar Th17 activity would occur with duvelisib. For duvelisib, we evaluated patients treated on our frontline study of duvelisib with FCR [4], in which 19 of 32 patients developed autoimmune toxicity. A subset of patients had early more severe toxicity, generally in cycle 2-3, while others developed only late toxicity during the duvelisib maintenance phase. We evaluated six patients with significant early toxicity, four patients without any toxicity and two patients with late toxicity, and again combined no toxicity with late toxicity for statistical evaluation. We assessed PBMCs serially drawn at screen timepoint (Scr), 7 days on duvelisib before starting FCR (C1), and at either 2 (*N* = 3) or 6 months (*N* = 9) post FCR (Post). As shown in Fig. 4A, B, respectively, we saw increased intracellular IL-17A and IL-17F in both CD4 and CD8 T cells at baseline comparing patients with toxicity and those with no or delayed toxicity, with IFNγ also increased particularly in CD8s. Although comparisons are limited by sample size, the effects on IL-17F appeared greater with duvelisib, whereas IL-17A and IFNγ were greater with idelalisib. Similar results were seen when evaluating the percentage of Th17-positive cells rather than the absolute number (Supplementary Fig. 4B). Interestingly, in contrast to our findings with idelalisib, no clear correlation of IL-17^+^ T cells with IGHV status was seen with duvelisib (Supplementary Fig. 5B). These differences could be related to PI3K gamma inhibition by duvelisib or alternatively to the FCR given in combination here; further studies will be required to validate these potential differences.

### Tissue-based activation of the Th17 pathway in vivo

**Fig. 5:**
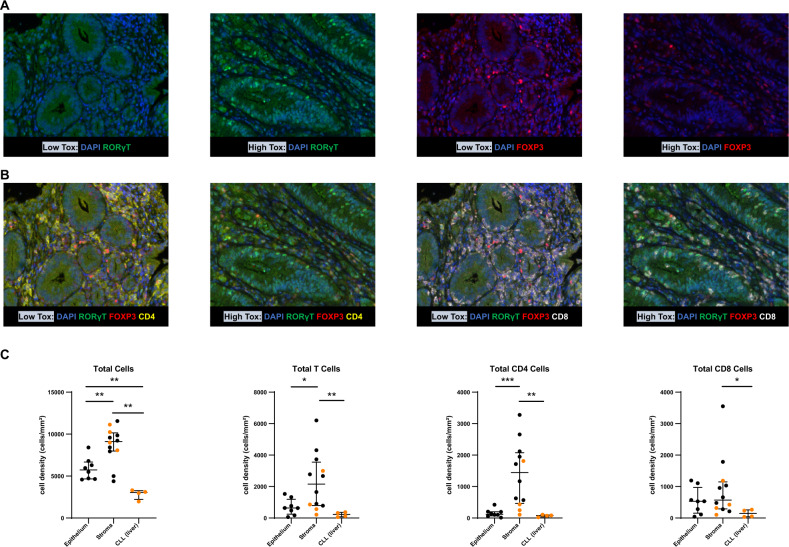
Multiplex immunofluorescence demonstrates tissue infiltration with CD4^+^ and CD8^+^ T Cells. **A** Representative images of colon with low and high toxicity demonstrating co-staining of RORγT cells and DAPI (left) or with FOXP3 and DAPI (right). **B** Representative images of colon with low and high toxicity demonstrating co-staining of CD4 (left) and CD8 (right) T cells with the other markers as above. **C** Quantification of cellular infiltration in each tissue compartment, showing epithelium, stroma and CLL infiltration in liver, measured by cell density. Represented are total cells, total T cells, total CD4 T cells and total CD8 T cells. The total cell graph includes CD4^+^ and CD8^+^ T cells plus other cells, including macrophages, fibroblasts, and normal epithelial cells. The total T cell graph includes only CD4^+^ and CD8^+^ T cells. Results from liver biopsies are in orange, while GI biopsies are in black. *p ≤ 0.05; **p ≤ 0.01; ***p ≤ 0.001.

Given these findings in peripheral blood, we were interested to determine whether we could validate activation of the Th17 pathway in vivo, in organs affected by PI3Kδ toxicity. We identified tissue biopsies done during each of these studies as part of clinical care and available for evaluation. We found 9 biopsies from high toxicity patients and 3 from low or no toxicity patients (Supplementary Table 2). Biopsies were GI (*n* = 8) or liver (*n* = 4), with epithelial and stromal compartments analyzed on GI biopsies, and tumor (infiltrating CLL) and liver/stromal compartments analyzed on liver biopsies. We sought to visualize, quantify, and analyze both Th17 cells, as measured by RORγt (Fig. 5A), and Tregs, as measured by FOXP3 staining (Fig. 5A), together with CD3, CD4 (Fig. 5B) and CD8-positive cells (Fig. 5B), in FFPE tissue biopsy samples comparing high to low/no toxicity. As shown in Fig. 5C, cellular infiltration was highest in stroma, followed by epithelium (Fig. 5C). No difference in overall cellular infiltration was seen based on whether or not the patient had significant toxicity (Supplementary Fig. 6). CD4 T cell infiltration was predominant in stroma, while CD8 cells were more evenly distributed across epithelium and stroma (Fig. 5C and Supplementary Fig. 6).

**Fig. 6:**
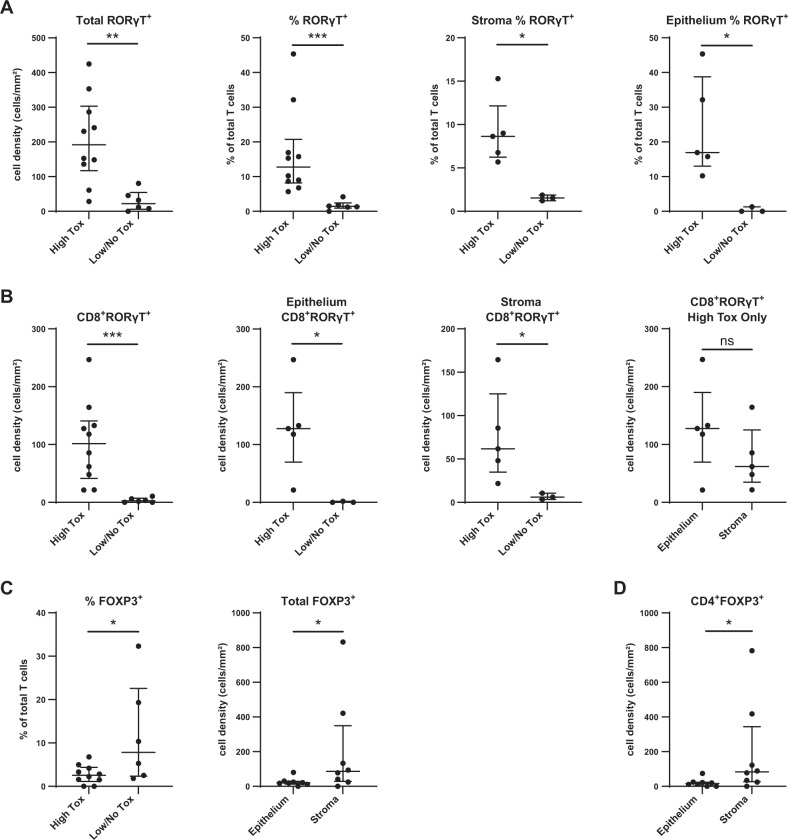
Multiplex immunofluorescence demonstrates infiltration by CD8^+^ RORγT^+^ T cells in GI biopsies from patients with high toxicity. **A** RORγT^+^ cells measured by cell density or % of total T cells, comparing patients with high GI toxicity to low/no toxicity. Infiltration is seen in both stroma and epithelium. **B** CD8^+^ RORγT^+^ infiltration in patients with high GI toxicity, seen in both stroma and epithelium. **C** FOXP3^+^ cells measured by % of total T cells or cell density, between patients with high GI toxicity or low/no toxicity. **D** CD4^+^ FOXP3^+^ cells measured by cell density between epithelium and stroma regions. **A**–**D** Patients with a liver biopsy are excluded from these graphs. ns = not significant; *p ≤ 0.05; **p ≤ 0.01; ***p ≤ 0.001.

To characterize the patterns of T cell infiltration, we analyzed the GI biopsies separately from the liver biopsies. In the GI biopsies, total RORγT^+^ cells were significantly higher in patients with toxicity compared to those without, by cell density, % of total cells and % of total T cells, and across both stroma and epithelium (Fig. 6A and Supplementary Fig. 7A). RORγT^+^ CD8^+^ T cells were enriched in the GI biopsies from high toxicity patients, across both stroma and epithelium (Fig. 6B). RORγT^+^ CD4^+^ T cells showed a trend to enrichment in the GI biopsies from high toxicity patients overall, with a significant increase in stroma in high toxicity patients (Supplementary Fig. 7B). Only four liver biopsies were available, all from patients with high toxicity, and they showed CD4 and CD8 T cell infiltration but did not show significance for RORγT^+^ cells (Supplementary Fig. 8).

### Tissue infiltration with Tregs is greater in patients without toxicity

We also evaluated tissue infiltration with Tregs in relation to autoimmune toxicity. In GI biopsies, FOXP3^+^ cells were enriched in total infiltrating T cells in patients without toxicity, particularly in stroma (Fig. 6C). Infiltration with CD4^+^ FOXP3^+^ cells was driving this finding (Fig. 6D). CD8^+^ FOXP3^+^ cells showed a similar but nonsignificant trend to enrichment in patients without toxicity, with more balanced distribution across epithelium and stroma (Supplementary Fig. 9).

## Discussion

Autoimmune toxicity of PI3K inhibitors remains unpredictable and sometimes severe, limiting their regular use when other options exist. Prior work in mouse models [7, 26] as well as our and others’ prior data in humans [2, 27] has suggested that loss of Tregs is the key component of this autoimmune toxicity. Here we demonstrate that both idelalisib and duvelisib inhibit Th1 and Treg differentiation in vitro but also promote Th17 differentiation. We further demonstrate that development of autoimmune toxicity in patients is associated with pre-existing expansion of Th17 T cells in peripheral blood, with increased tissue infiltration in patients with active autoimmune complications of PI3Kδ inhibitors, at least in intestinal tissue. These same tissues show depletion of Tregs as compared to CLL patients who do not develop toxicity, suggesting that PI3K inhibitor-induced loss of Tregs may unleash the pathologic activity of pre-existing Th17 cells, leading to autoimmune toxicity. Overall, the patterns are similar in both these previously untreated and our previously reported relapsed patients [16].

Our results demonstrate that both idelalisib and duvelisib have potent effects on T cells. They both substantially inhibit T cell proliferation, consistent with prior work using PI3Kδ-kinase dead mouse models [7]. Interestingly, duvelisib is a more potent inhibitor of proliferation than idelalisib; whether related to more potent inhibition of the delta isoform, or to gamma isoform inhibition, is not known. We also demonstrate that both drugs impair Th1 and Treg differentiation substantially, while enhancing Th2 and Th17 differentiation. The impact of idelalisib and duvelisib on Treg differentiation and function in vitro and in mice has been previously described [27, 28], and our data are consistent with those findings. We have also demonstrated the effect of idelalisib on circulating Tregs in vivo in patients [2] and here expand that finding to show that Tregs are reduced in human tissues from patients with autoimmune toxicity of PI3Kδ inhibitors. This loss of Tregs may be permissive for the development of autoimmune toxicity in the context of Th17 activation.

The impact of idelalisib and duvelisib on Th17 differentiation in vitro has not to our knowledge been previously described, but was suggested by our T cell findings from CyTOF in the clinical trials described here [15, 16, 19, 22]. A shift toward Th17 cytokine bias has also been reported in the colitis that develops in mice harboring a kinase-dead p110δ [29]. Prior work demonstrates that the Th17 pathway is more active in CLL patients compared to healthy controls, and furthermore has suggested that autologous CLL B cells and the tumor microenvironment promote Th17 differentiation [25, 30]. These observations together with our current findings suggest that CLL patients may be at greater risk of autoimmune toxicity compared to other lymphoma patients, if they have higher IL-17A-positive T cells at baseline. Anecdotal cross-trial comparisons of the incidence of toxicity appear to support that observation [2, 3, 31], but further data in other patient populations are needed.

We extend our in vitro findings to demonstrate that CD4 and CD8 T cells show increased baseline Th17 differentiation in CLL patients with early fulminant autoimmune toxicity compared to those without toxicity, and even compared to those with more delayed toxicity. Whether the mechanism of early fulminant toxicity differs from that of more delayed toxicity is not known. Limited evaluable samples not exposed to steroids or extended drug holds reduced our ability to evaluate patients with delayed toxicity. It is reasonable to hypothesize that some patients are more immunologically primed for toxicity, perhaps by having increased baseline Th17 T cells, or alternatively by having fewer Tregs or a greater drop with the initiation of PI3Kδ inhibitor, while in those less primed, the accumulating exposure to drug may result in similar immunologic changes over time. In this study we do not see obvious increase in Th17 T cells over time, but further investigation of later timepoints in patients who develop late toxicity, also evaluating delayed declines in Tregs, is warranted. The differences observed here between idelalisib and duvelisib also require confirmation, particularly as the impact of FCR on the T cells in the duvelisib study is unknown. Further investigation to establish absolute baseline levels of Th17 T cells and Tregs that are associated with risk of greater toxicity would also be beneficial as it would allow patient screening prior to therapy.

Finally, we demonstrate that in patients treated with PI3Kδ inhibitors, tissues with autoimmune inflammation show increased RORγT-positive T cells and decreased Tregs, compared to patients with low or no toxicity, consistent with the CyTOF data and the peripheral blood findings. Taken together these data strongly support a Th17:Treg shift as the major contributor to these autoimmune toxicities.

Our data remain limited by small patient numbers for study. The low absolute numbers and low percentages of Th17s in blood compared to other studies may be due to sensitivity to cell destruction following freezing and/or to the need to match the number of cells per sample for analysis. However, the consistency across all patients and similar freezing techniques suggests that the relative differences remain significant. The results are also supported by the multiplex immunofluorescence studies and by the IFNγ-positive cells which as expected are in much higher numbers.

Recent data has shown that autoimmune toxicity correlates with improved survival in patients on PI3K inhibitors [32], raising the possibility that improved anti-tumor immunity may be induced by these T cell changes, as previously described in mouse models [7]. Understanding the mechanisms underlying toxicity as well as antitumor immunity with PI3Kδ inhibitors provides an exciting path forward for future work to focus on controlling pro-inflammatory Th17 responses that lead to toxicity, while allowing Treg reduction to release anti-tumor immunity and enhance tumor control. These efforts may hopefully allow us to realize the full potential of PI3Kδ inhibition in malignant disease.

## Supplementary information

Supplementary Methods, Tables, and Figures

## Data Availability

The datasets generated during and/or analyzed during the current study are available from the corresponding author on reasonable request.
