# Supplementary material for: Circulating Th17 T cells at treatment onset predict autoimmune toxicity of PI3Kδ inhibitors
Source: Blood Cancer J. 2023 Feb 2;13(1):22. doi: 10.1038/s41408-023-00788-9 (PMC9895075; doi:10.1038/s41408-023-00788-9)
Supplement: Supplementary file 1 — Supplementary Methods, Tables, and Figures [file 41408_2023_788_MOESM1_ESM.pdf]

Supplementary Materials

Supplementary Methods
Supplementary Table 1. Patient Characteristics
Supplementary Table 2. Samples for Multiplex Immunofluorescence
Supplementary Figure 1. Flow cytometry gating strategy for quantifying percentages of total IL-17A+ and total IL-17F+ cells.
Supplementary Figure 2. Flow cytometry gating strategy for quantifying percentages of CD4+ and CD8+ cell populations expressing IL-17A, IL-17F or IFNγ alone or in combination.
Supplementary Figure 3. Quantification of % Th1 and Th2 differentiation, comparing DMSO treated cells to either idelalisib or duvelisib. *$p \leq 0.05$.
Supplementary Figure 4. **A.** Idelalisib % Th17 comparing early toxicity (Tox) to either delayed or no toxicity (No Tox). **B.** Duvelisib % Th17 comparing early toxicity (Tox) to either delayed or no toxicity (No Tox). Samples prior to therapy: C1 in **A**; Scr in **B**. *$p \leq 0.05$; **$p \leq 0.01$.
Supplementary Figure 5. Comparison of absolute number of CD4 and CD8 T cells with Th17 differentiation, by IGHV mutation status. **A**. Idelalisib cohort. **B**. Duvelisib cohort. *$p \leq 0.05$; **$p \leq 0.01$.
Supplementary Figure 6. Total cellular infiltration and T cell infiltration comparing epithelium, stroma, and regions of CLL in liver, between patients with high toxicity or low/no toxicity. Liver biopsy results are highlighted in orange. *$p \leq 0.05$; **$p \leq 0.01$.
Supplementary Figure 7. RORγT+ infiltrating cells in biopsies from patients with high toxicity compared to low/no toxicity. **A**. Total cells and enrichment in both stroma and epithelium. **B.** CD4+RORγT+ cells, comparing high toxicity and low/no toxicity patients. Patients with a liver biopsy are excluded from these graphs. ns = not significant; *$p \leq 0.05$; ***$p \leq 0.001$.
Supplementary Figure 8. Cell density in liver biopsy patients, comparing stroma and CLL regions. All four patients with liver biopsies had high toxicity. ns = not significant; *$p \leq 0.05$.
Supplementary Figure 9. Comparison of CD8+FOXP3+ cells and CD4+FOXP3+ cells in biopsies from patients with high toxicity compared to low/no toxicity. Patients with a liver biopsy are excluded from these graphs. ns = not significant.

Supplementary Methods

Proliferation assay

Frozen healthy PBMCs were thawed, washed and resuspended at a concentration of 2 million cells per mL. The PBMCs were then labeled using the cell division tracker dye CellTrace Violet (ThermoFisher Scientific) and stimulated with anti-CD3/CD28 T cell activation beads (Dynabeads) at 4 million beads per mL at a 1:1 cell:bead ratio for 72 hours in the presence or absence of drug treatment (idelalisib or duvelisib at 1 or 5 μM, chosen based on the 1 μM achieved drug concentration in patients in trials). Cells were stained with CD3-APC-Cy7 prior to flow cytometry. Proliferation was determined by assessing CellTrace Violet dye dilution by flow cytometry (BD LSR Fortessa instrument) on CD3 gated cells. The proliferation index was calculated using the ModFit LT algorithm to quantify cell divisions.

Differentiation assay

Whole blood collars from healthy donors were obtained from the Crimson Core at Brigham and Women's Hospital and PBMCs were isolated using Ficoll centrifugation. Naïve CD4+ T cells were isolated from these healthy PBMCs using a STEMCELL Technologies kit (#19555) and resuspended at a concentration of 2 million cells per mL. Naïve CD4+ T cell purity was determined by flow cytometry using CD4, CD25, CD45RA and CD45RO antibodies, with over 98% purity achieved by analysis of the CD4+ CD25+ CD45RA+ CD45RO- population. We differentiated naïve CD4+ T cells toward a Treg, Th17, Th1 and Th2 phenotype in the presence of CD3/CD28 Dynabeads at 4 million beads per mL at a 1:1 cell:bead ratio (Thermo Fisher #11141D) and 10 μM duvelisib/idelalisib or DMSO using CellXVivo differentiation kits from R&D systems. Experimental setup was done as per kit instructions. Treg differentiation was evaluated by measuring intracellular FOXP3 staining at day 7, Th17 differentiation was determined by RORγT intracellular staining at day 10, Th1 differentiation by using T-bet intracellular staining at day 5 and Th2 differentiation by using GATA3 intracellular staining at day 13 using flow cytometry (BD LSR Fortessa instrument).

Image Acquisition and Cell Identification

Image acquisition was performed using the Mantra multispectral imaging platform (PerkinElmer, Hopkinton, MA). Areas with non-tumor or residual normal tissue were excluded from the analysis. Representative regions of interest were chosen by the pathologist, and 3-5 fields of view (FOVs) were acquired at 20x resolution as multispectral images. After image capture, the FOVs were spectrally unmixed and then analyzed using supervised machine learning algorithms within Inform 2.4 (PerkinElmer). Thresholds for positive staining and the accuracy of phenotypic algorithms were optimized and confirmed by the pathologist (S.J.R.) for each case.

Supplementary Table 1

	Idelalisib Cohort	Duvelisib Cohort	Histopathology Cohort
**Number of patients**	**19**	**12**	**11**
**% Male**	**79%**	**75%**	**55%**
**% Unmutated IGHV (≥98% IGHV homology)**	**53%**	**58%**	**36%**
**Karyotype Abnormalities**			
**0-2 Aberrations**	**13/16**	**12/12**	**7/10**
**3-4 Aberrations**	**1/16**	**0/12**	**1/10**
**5+ Aberrations**	**2/16**	**0/12**	**2/10**
**FISH Abnormalities**			
**Normal**	**4/19**	**4/12**	**7/11**
**13q deletion**	**8/19**	**2/12**	**2/11**
**11q deletion**	**1/19**	**3/12**	**0/11**
**17p deletion**	**3/19**	**1/12**	**1/11**
**Trisomy 12**	**3/19**	**2/12**	**1/11**
**TP53 mutation**	**4/16**	**0/12**	**2/9**
**NOTCH1 mutation**	**3/19**	**1/12**	**1/11**
**Median age at diagnosis**	**64**	**51**	**56**
**Median age at treatment initiation**	**70**	**54**	**59**
**Median time from diagnosis to study therapy (months)**	**37**	**12**	**49**
**Median time from start of study therapy to toxicity (months)**	**1**	**6**	**1**
**% of patients with high toxicity**	**79%**	**42%**	**82%**
**% of patients with severe tox due to transaminitis**	**80%**	**100%**	**89%**
**% of patients in Histopath cohort on idelalisib**			**55%**
**% of patients in Histopath cohort that overlap with the other 2 cohorts**			**64%**

Supplementary Table 2

Protocol	Patient Number	Biopsy	Tox
13-309	1	gastric	High Tox
13-309	2	sigmoid colon	High Tox
13-309	3	enteroscopy	Low Tox
13-309	3	colon	Low Tox
13-309	4	colon	High Tox
13-309	5	liver	High Tox
13-309	6	liver	High Tox
14-193	7	colon	High Tox
14-193	8	sigmoid colon	High Tox
14-193	9	colon	No Tox
14-193	10	liver	High Tox
14-193	11	liver	High Tox
control		normal liver	None
control		normal liver	None
control		liver bx with CLL	None
control		liver bx with CLL	None
control		colon cancer	None
control		colon cancer	None
control		colitis	None
control		colitis	None
control		colitis	None
control		tonsil	None

Supplementary Figure 1

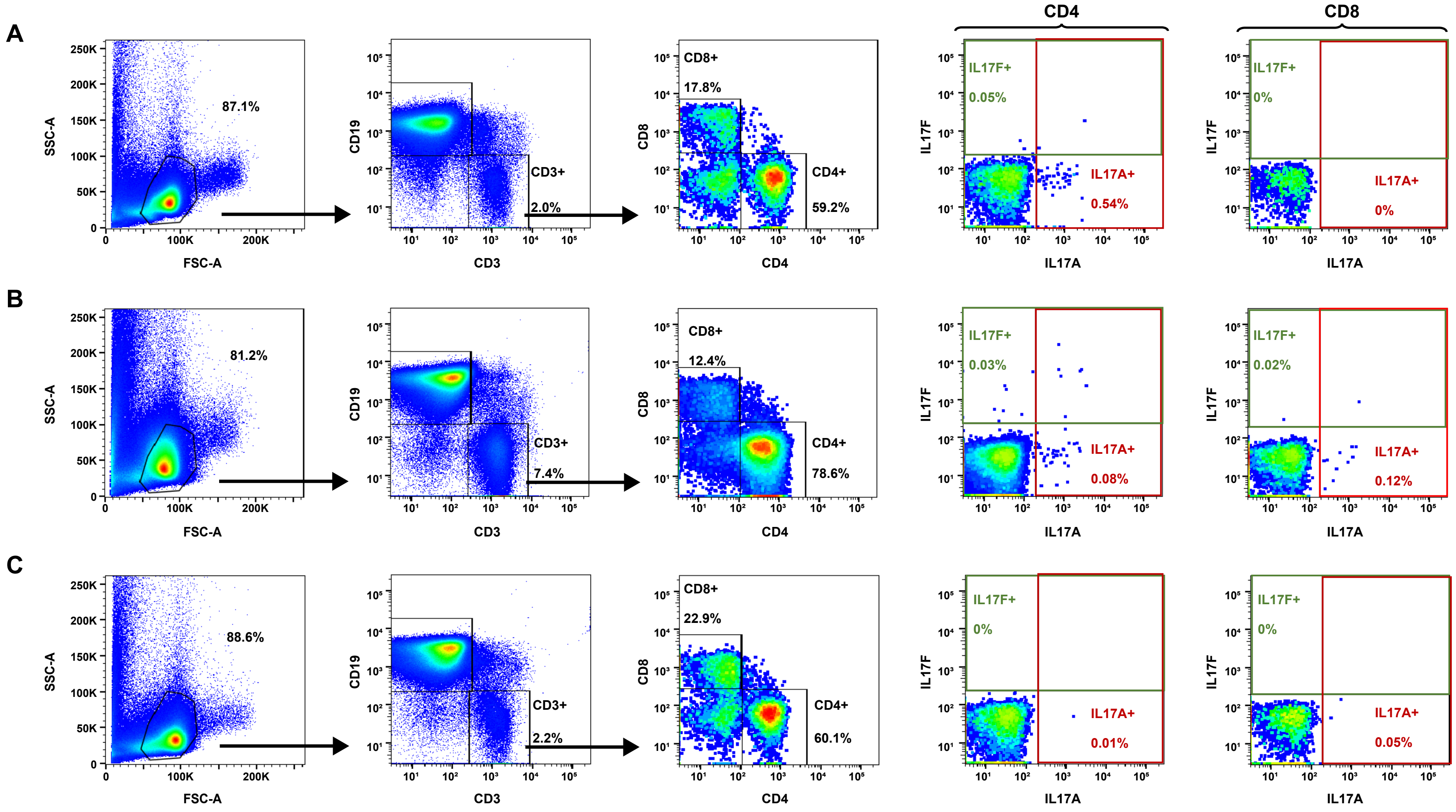

Supplementary Figure 1. Flow cytometry gating strategy for quantifying percentages of total IL-17A+ and total IL-17F+ cells. Frozen aliquots of peripheral blood mononuclear cells isolated from patients treated with idelalisib or duvalisib were thawed, stimulated for 3 hours with PMA and ionomycin, and then stained for surface markers (CD3, CD4, CD8 and CD19) and intracellular cytokines IL-17A and IL-17F) as described in Methods. Representative plots showing gating strategy for quantifying percentages of total IL-17A+ and total IL-17F+ CD4 and CD8 cells for (**A**) a patient with high percentage of CD4+IL-17A+ cells, (**B**) a patient with high percentage of CD8+IL-17A+ cells, and (**C**) a patient with low percentage of CD4+IL-17A+ and CD8+IL-17A+ cells are shown.

Supplementary Figure 2

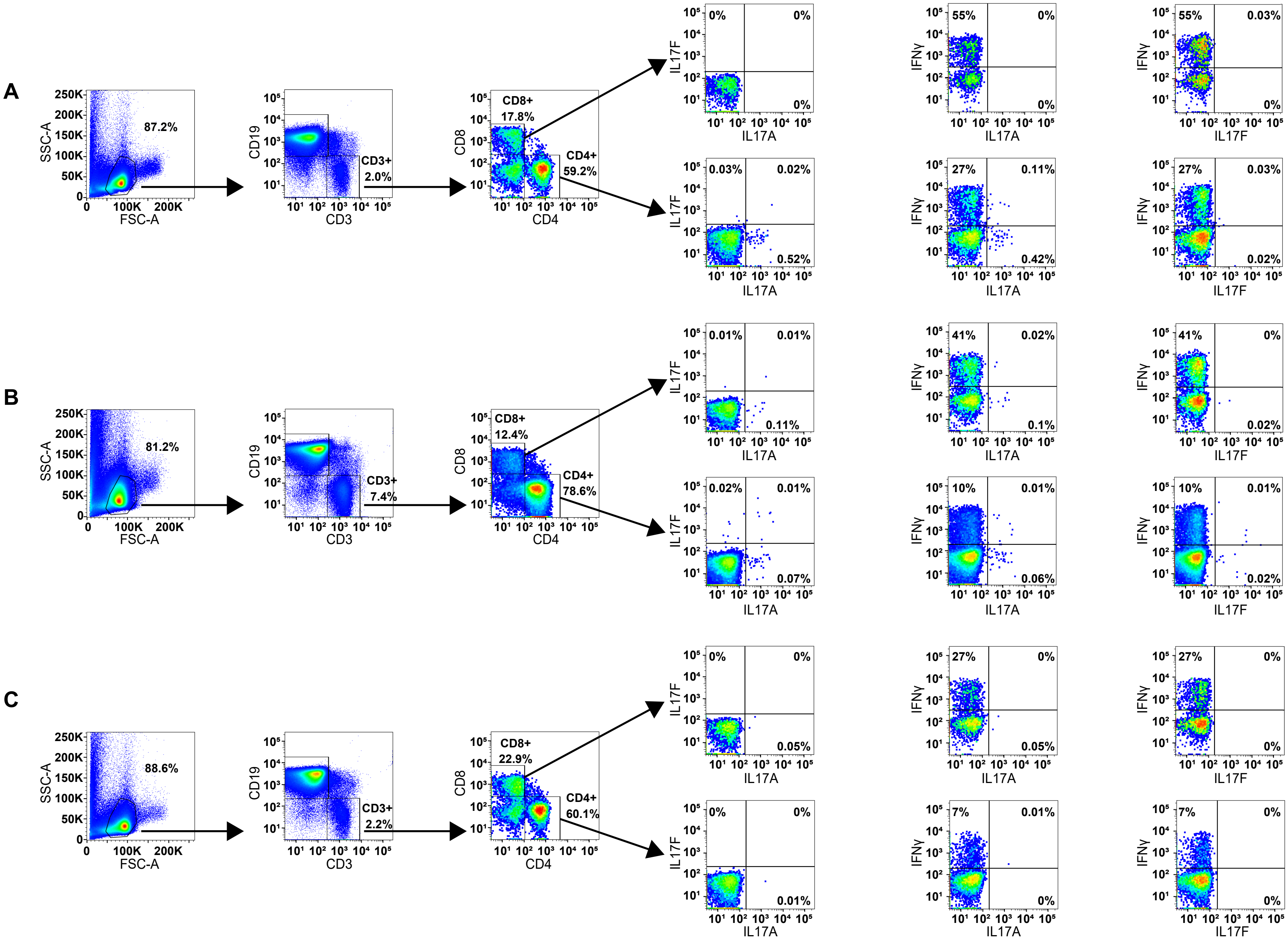

Supplementary Figure 2. Flow cytometry gating strategy for quantifying percentages of CD4+ and CD8+ cell populations expressing IL-17A, IL-17F or IFNγ alone or in combination. Frozen aliquots of peripheral blood mononuclear cells isolated from patients treated with idelalisib or duvelisib were thawed, stimulated for 3 hours with PMA and ionomycin, and then stained for surface markers (CD3, CD4, CD8 and CD19) and intracellular cytokines IL-17A, IL-17F and IFNγ) as described in Methods. Representative plots showing gating strategy for quantifying percentages of CD4+ and CD8+ cell populations expressing IL-17A+, IL-17F+, or IFNγ alone or in combination for (**A**) a patient with high percentage of CD4+IL-17A+ cells, (**B**) a patient with high percentage of CD8+IL-17A+ cells, and (**C**) a patient with low percentage of CD4+IL-17A+ and CD8+IL-17A+ cells are shown.

Supplementary Figure 3

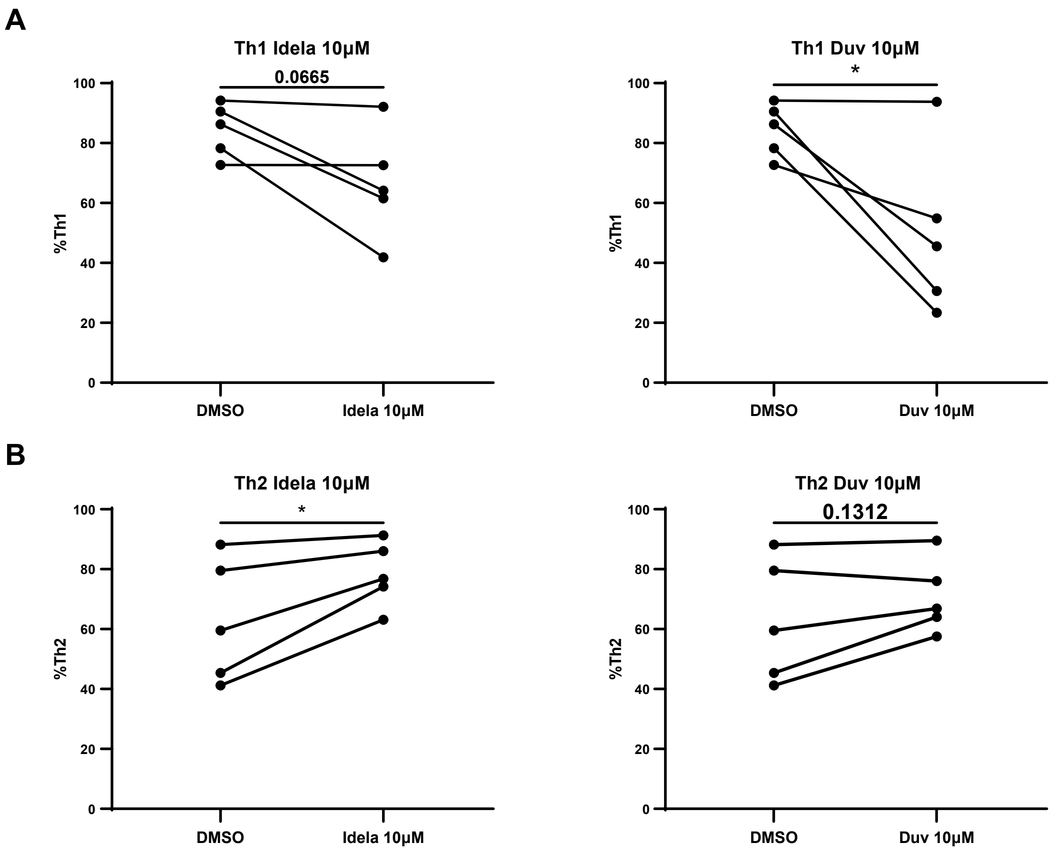

Supplementary Figure 3. Quantification of % Th1 and Th2 differentiation, comparing DMSO treated cells to either idelalisib or duvelisib. *$p \leq 0.05$.

Supplementary Figure 4

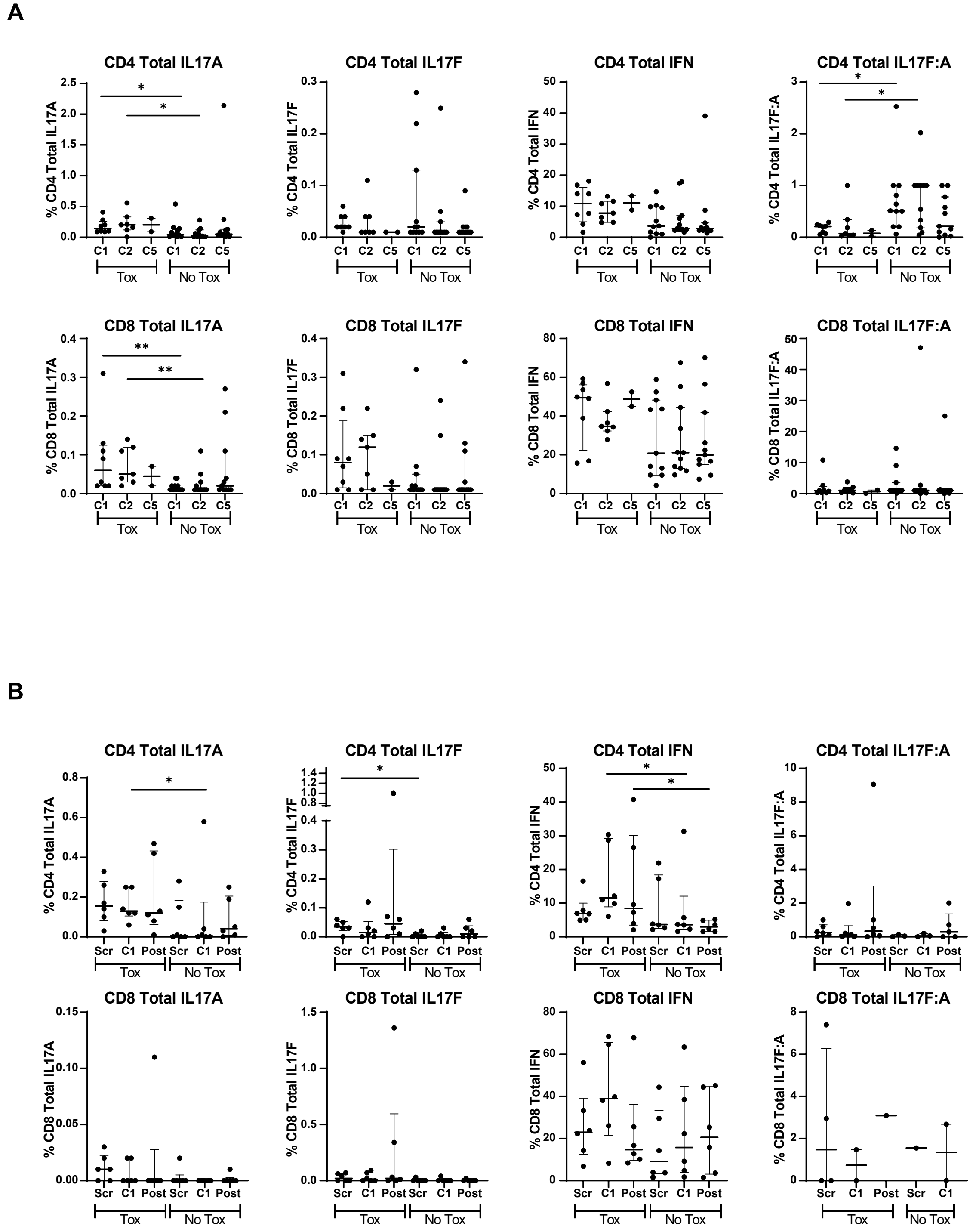

Supplementary Figure 4. A. Idelalisib % Th17 comparing early toxicity (Tox) to either delayed or no toxicity (No Tox). **B.** Duvelisib % Th17 comparing early toxicity (Tox) to either delayed or no toxicity (No Tox). Samples prior to therapy: C1 in A; Scr in B. *p ≤ 0.05; **p ≤ 0.01.

Supplementary Figure 5

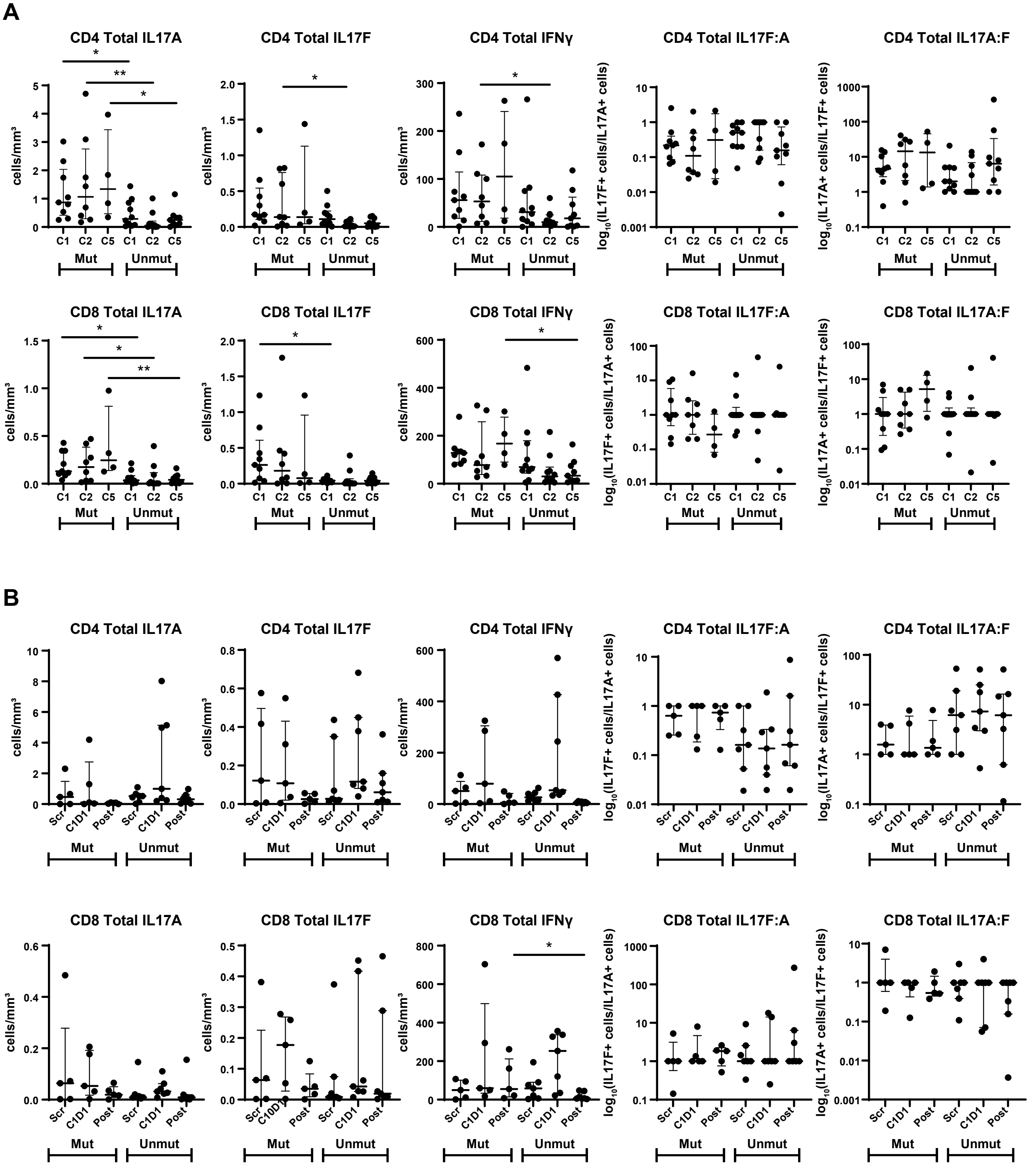

Supplementary Figure 5. Comparison of absolute number of CD4 and CD8 T cells with Th17 differentiation, by IGHV mutation status. **A.** Idelalisib treatment. **B.** Duvelisib treatment. *p ≤ 0.05; **p ≤ 0.01.

Supplementary Figure 6

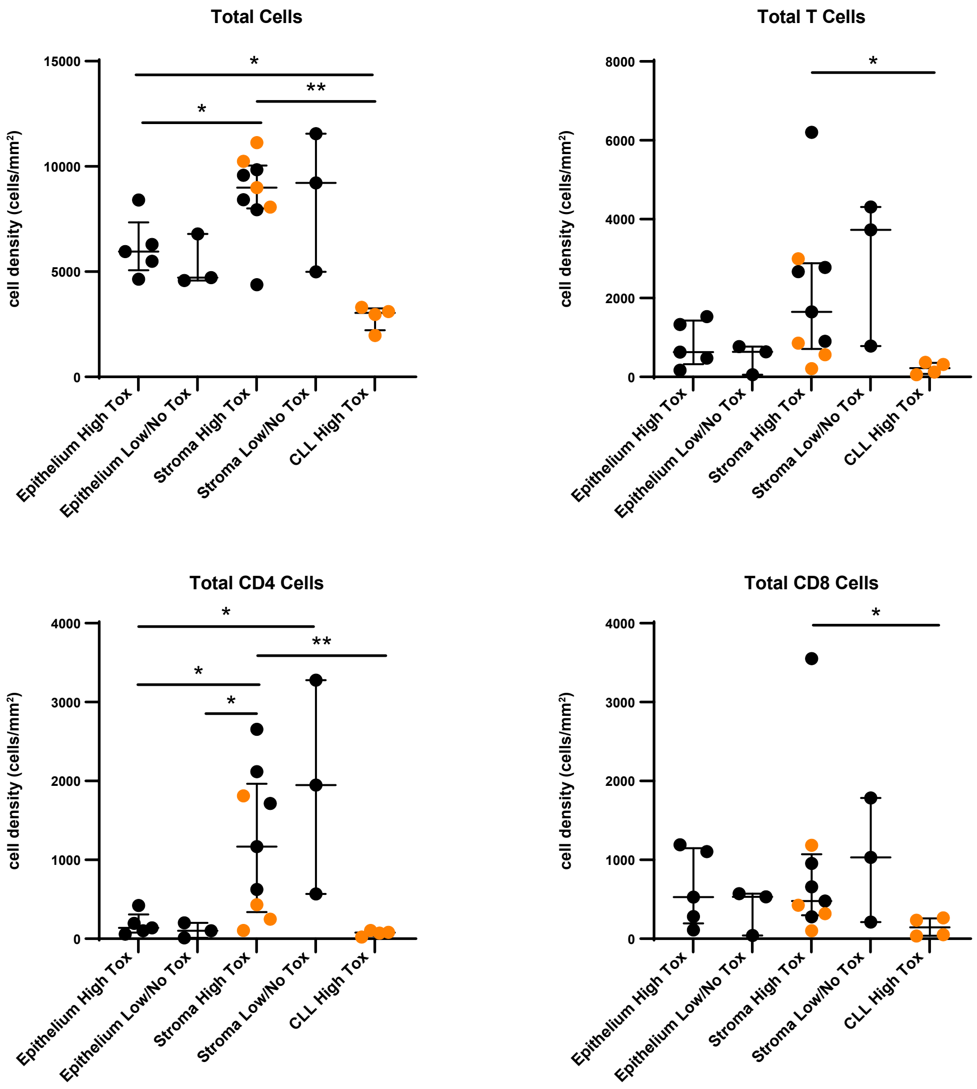

Supplementary Figure 6. Total cellular infiltration and T cell infiltration comparing epithelium, stroma, and regions of CLL in liver, between patients with high toxicity or low/no toxicity. Liver biopsy results are highlighted in orange. *p ≤ 0.05; **p ≤ 0.01.

Supplementary Figure 7

A

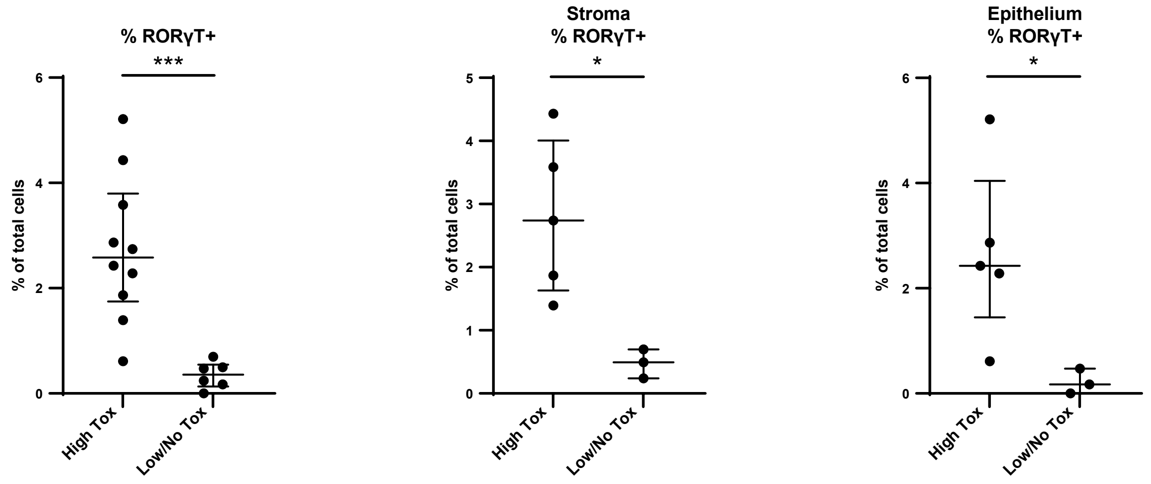

B

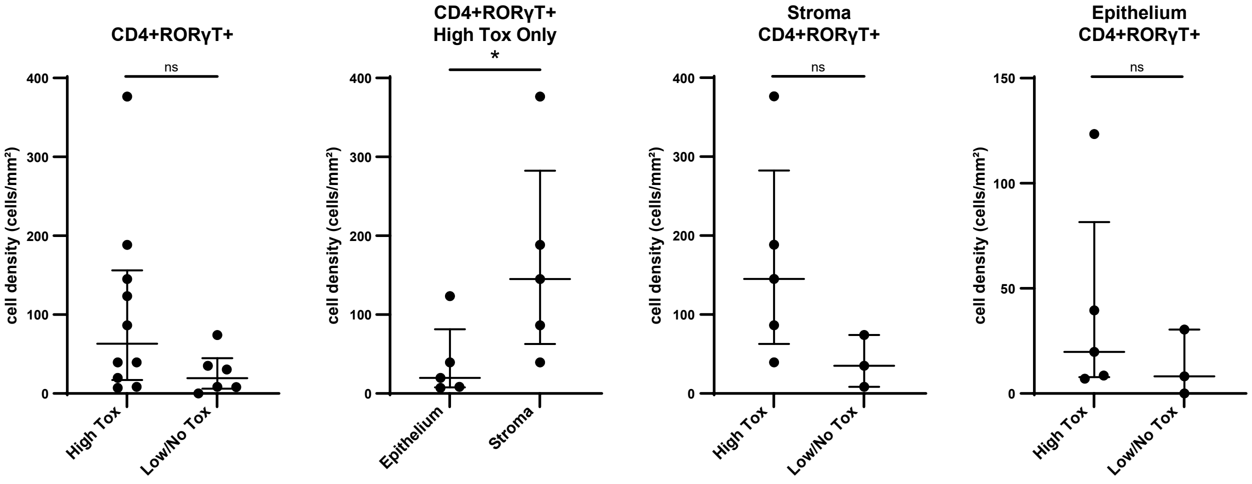

Supplementary Figure 7. RORγT+ infiltrating cells in biopsies from patients with high toxicity compared to low/no toxicity. **A.** Total cells and enrichment in both stroma and epithelium. **B.** CD4+RORγT+ cells, comparing high toxicity and low/no toxicity patients. Patients with a liver biopsy are excluded from these graphs. ns = not significant; *p ≤ 0.05; ***p ≤ 0.001.

Supplementary Figure 8

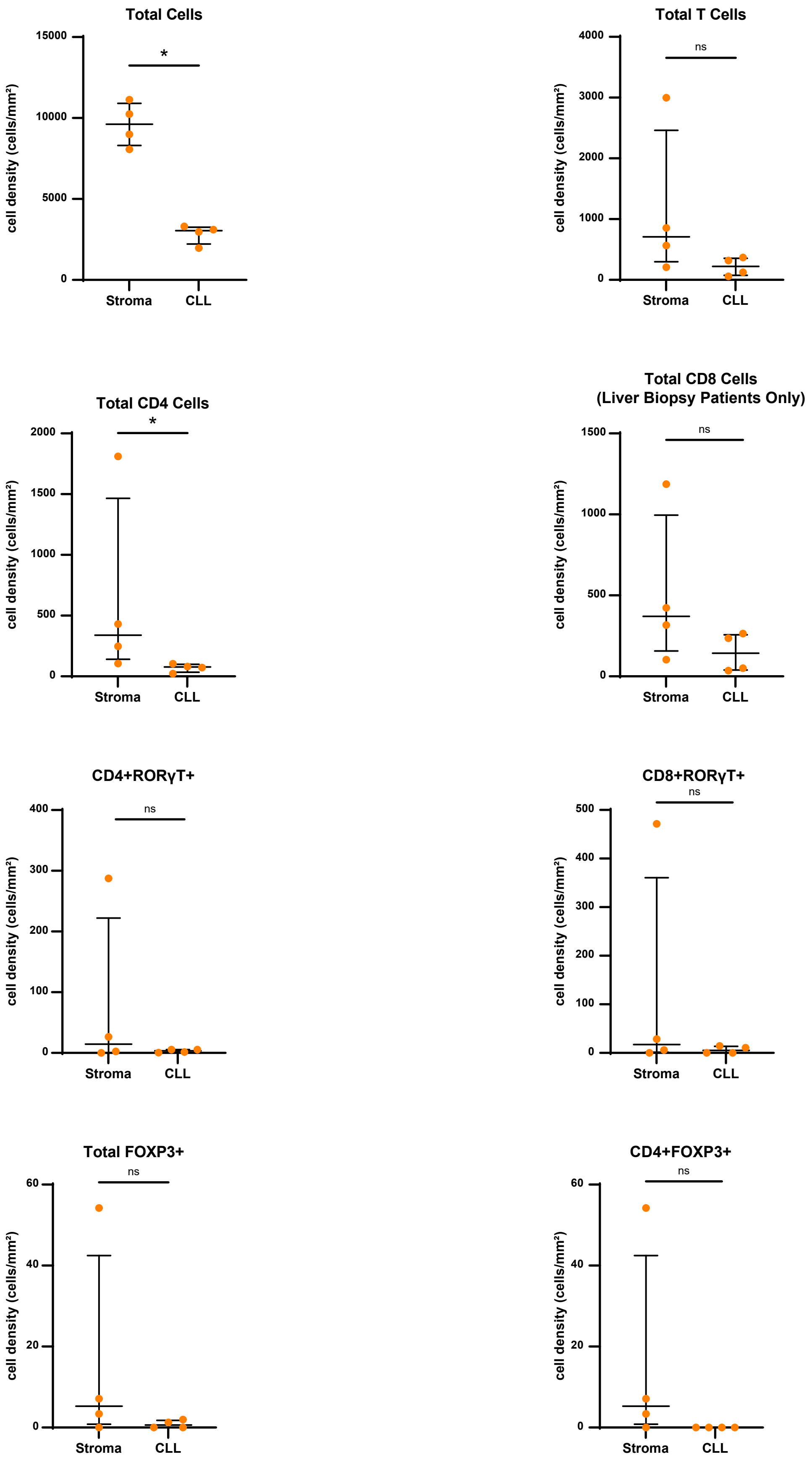

Supplementary Figure 8. Cell density in liver biopsy patients, comparing stroma and CLL regions. All four patients with liver biopsies had high toxicity. ns = not significant; *$p \leq 0.05$.

Supplementary Figure 9

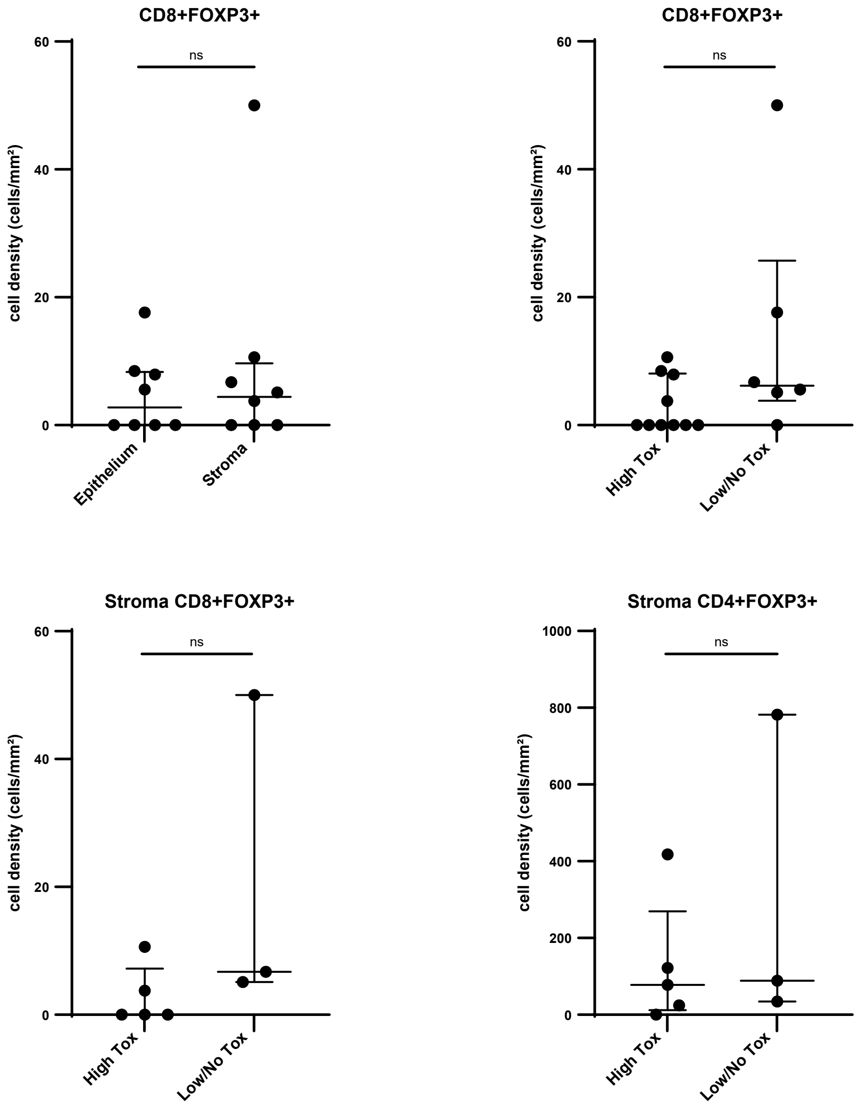

Supplementary Figure 9. Comparison of CD8+FOXP3+ cells and CD4+FOXP3+ cells in biopsies from patients with high toxicity compared to low/no toxicity. Patients with a liver biopsy are excluded from these graphs. ns = not significant.
